# CRISPR-based tools for targeted genetic manipulation in pathogenic *Sporothrix* species

**DOI:** 10.1128/spectrum.05078-22

**Published:** 2023-09-14

**Authors:** Remi Hatinguais, Ian Leaves, Gordon D. Brown, Alistair J. P. Brown, Matthias Brock, Roberta Peres da Silva

**Affiliations:** 1 Medical Research Council Centre for Medical Mycology, University of Exeter, Exeter, United Kingdom; 2 Fungal Biology Group, School of Life Sciences, University of Nottingham, Nottingham, United Kingdom; Universidade de Brasilia, Brasilia, Brazil

**Keywords:** *Sporothrix*, DHN-melanin, *pks1*, *ku80*, CRISPR/Cas9

## Abstract

*Sporothrix brasiliensis* is an emerging fungal pathogen frequently associated with zoonotic transmission of sporotrichosis by contaminated cats. Within 25 years, the disease has spread not only throughout Brazil but now to neighboring countries in Latin America. Thermo-dimorphism, melanin, glycans, adhesins, and secreted vesicles have been associated with the ability of *Sporothrix* species to cause disease in the mammalian host. Although certain virulence factors have been proposed as potential determinants for sporotrichosis, the scarcity of molecular tools for performing reverse genetics in *Sporothrix* has significantly impeded the dissection of mechanisms underlying the disease. Here, we demonstrate that PEG-mediated protoplast transformation is a powerful method for heterologous gene expression in *S. brasiliensis, S. schenckii,* and *S. chilensis*. Combined with CRISPR/Cas9 gene editing, this transformation protocol enabled the deletion of the putative DHN-melanin synthase gene *pks1*, which is a proposed virulence factor of *Sporothrix* species. To improve *in locus* integration of deletion constructs, we deleted the KU^80^ homolog that is critical for non-homologous end-joining DNA repair. The use of Δ*ku80* strains from *S. brasiliensis* enhanced homologous-directed repair during transformation resulting in increased targeted gene deletion in combination with CRISPR/Cas9. In conclusion, our CRISPR/Cas9-based transformation protocol provides an efficient tool for targeted gene manipulation in *Sporothrix* species.

**IMPORTANCE:**

Sporotrichosis caused by *Sporothrix brasiliensis* is a disease that requires long periods of treatment and is rapidly spreading across Latin America. The virulence of this fungus and the surge of atypical and more severe presentations of the disease raise the need for an understanding of the molecular mechanisms underlying sporotrichosis, as well as the development of better diagnostics and antifungal therapies. By developing molecular tools for accurate genetic manipulation in *Sporothrix,* this study addresses the paucity of reliable and reproducible tools for stable genetic engineering of *Sporothrix* species, which has represented a major obstacle for studying the virulence determinants and their roles in the establishment of sporotrichosis.

## INTRODUCTION

Sporotrichosis is a neglected fungal disease caused by thermo-dimorphic fungi belonging to the *Sporothrix* complex, which includes *S. brasiliensis*, *S. schenckii sensu stricto*, *S. luriei,* and *S. globosa*. While those species are frequently associated with human and animal disease, the environmental species *S. mexicana*, *S. pallida*, and *S. chilensis* are referred to as opportunistic pathogens that cause infections in immunocompromised patients (1, 2). Sporotrichosis is classically transmitted by traumatic inoculation of fungal propagules into subcutaneous tissue (3). Nevertheless, ongoing outbreaks in Brazil are mainly related to the epizootic transmission among cats infected with *S. brasiliensis,* a species of high virulence (4). Due to their close proximity to humans, zoonotic transmission from cats via scratches or bites has become a challenge for confining the geographical advance of the disease (1 – 5). Human and feline sporotrichosis outbreaks were initially observed in the state of Rio de Janeiro about 25 years ago. Outbreaks are now observed throughout almost all of Brazil and in neighbouring Latin American countries, such as Argentina and Chile (2, 6, 7), and at least three cases of human sporotrichosis, transmitted by an imported infected cat, have now been reported in the United Kingdom (8, 9). Thermo-dimorphism, glycans, adhesins, secreted vesicles, and melanin have all been associated with the ability of the fungus to cause disease in the mammalian host (5, 10). However, their contributions to virulence remain to be confirmed experimentally.

*S. brasiliensis* and *S. schenckii* possess three pathways related to melanin production: 3,4-dihydroxy-l-phenylalanine (l-DOPA), 1,8-dihydroxynaphthalene (DHN)-melanin, and pyomelanin (11, 12). The presence of melanin in fungal cells has been related to resistance to environmental stresses and the survival of the pathogen during host interactions (12 – 14). UV-induced DHN-melanin-deficient strains of *S. schenckii* were less resistant to oxidative/nitrosative stresses and phagocytosis by human monocytes or murine macrophages (15). In addition, DHN-melanin inhibitors enhanced the *in vitro* susceptibility of *S. brasiliensis* and *S. schenckii* to the antifungal drug terbinafine. Furthermore, yeast cells from *S. brasiliensis* and *S. schenckii* grown in the presence of the melanin-inducing l-DOPA or l-tyrosine were less susceptible to amphotericin B but not itraconazole (16, 17). In contrast, the inhibition of the eumelanin or pyomelanin pathways did not result in an increased antifungal susceptibility (17). This indicates a general importance of all three types of melanin during host infection and the particular importance of DHN-melanin in promoting resistance to antifungal therapy.

In the last two decades, there has been an increased effort to develop new tools for diagnosis, epidemiologic, and virulence studies, but few studies have focused on new approaches to improve the molecular and genetic manipulation of the *Sporothrix* species complex (2, 18). Currently, the genetic manipulation of *Sporothrix* species has focused on gene silencing using vectors delivered to fungal cells *via Agrobacterium tumefaciens*-mediated transformation or by random UV-induced mutation of *S. schenckii* and *S. globosa* (15, 19 – 23). *Sporothrix* species have represented a challenge for the application of universal genetic engineering tools (24). This has hindered the elucidation of specific mechanisms that promote the survival and immune evasion of *Sporothrix* species during host interactions.

In this study, we established a protocol for accurate gene disruption in *S. brasiliensis* and *S. schenckii* using the CRISPR/Cas9 system. Furthermore, we standardized a transformation protocol for ectopic integration and heterologous gene expression in *S. chilensis,* a species without a sequenced genome. Moreover, we show that the deletion of the *ku80* gene, together with the functional expression of a red-shifted luciferase in *S. brasiliensis* strains, facilitates the screening of transformants and provides a promising tool for *in vivo* real-time monitoring of sporotrichosis.

## RESULTS

### Protoplast-mediated transformation grants delivery and ectopic integration of synthetic genes in pathogenic *Sporothrix* species

**Fig 1:**
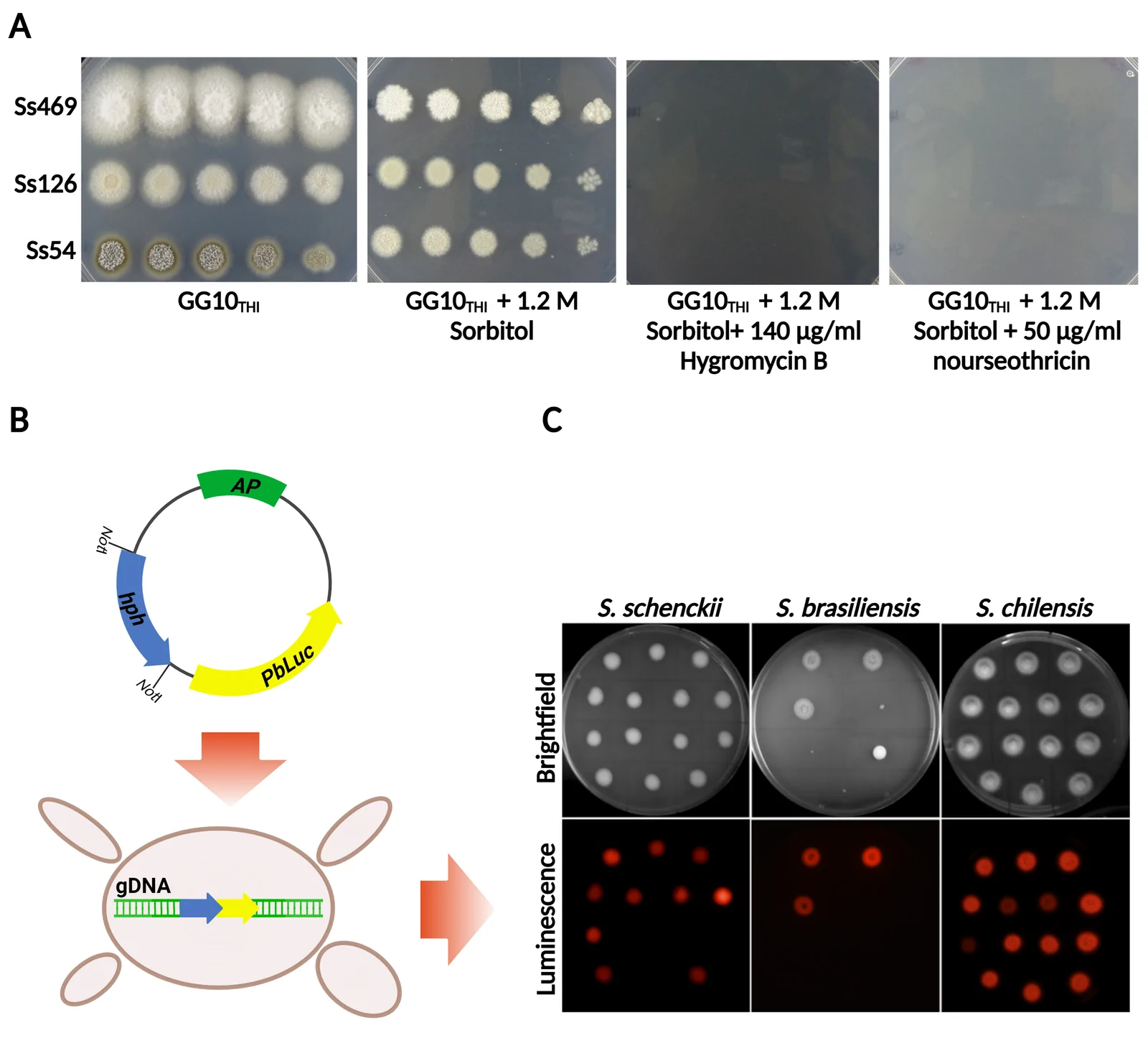
Protoplast-mediated transformation of *S. schenckii, S. brasiliensis,* and *S. chilensis*. (**A**) Spot assays of five serial dilutions ranging from 3 × 10^5^ to 30 conidia from *S. brasiliensis* Ss54*, S. schenckii* Ss126, and *S. chilensis* Ss469 on GG10_THI_, GG10_THI_ supplemented with 1.2 M sorbitol, 140 µg/mL hygromycin B, and 50 µg/mL nourseothricin. (**B**) Schematic representation of the plasmid employed in the transformation protocol showing the *hph* resistance cassette and red-shifted firefly luciferase (*PbLuc*) construct. (**C**) Imaging of transformants containing the luciferase construct inoculated onto agar plates containing Hygromycin B (140 µg/mL) and d-luciferin (0.2 mM). Brightfield (upper panels) and luminescence (bottom panels) were recorded after 7 days of incubation.

Protoplast-mediated transformation of filamentous fungi is a well-established method (25), but such an approach has not been explored for its suitability on the mycelium phase of thermo-dimorphic fungi, such as *Sporothrix* species. To develop an efficient protocol for transformation in *Sporothrix* species, our first step was to assess appropriate combinations of growth medium and selection markers that could be utilized during polyethylene glycol (PEG)-mediated protoplast transformation. We performed spot assays of serial dilutions of conidia from the strains *S. brasiliensis* Ss54*, S. schenckii* Ss126, and *S. chilensis* Ss469. These strains were tested for their ability to grow in modified *Aspergillus* minimal medium containing 50 mM glucose as the carbon source and 10 mM glutamine as the nitrogen source (GG10) (26) and supplemented with thiamine (GG10_THI_). Sorbitol was added as an osmotic stabilizer of protoplasts. In addition, we tested three different concentrations of antibiotics for two selection markers previously utilized in *S. schenckii* transformation (20, 22): hygromycin B (80, 140, and 180 µg/mL) and nourseothricin (50, 100, and 200 µg/mL). The concentrations of 140 µg/mL hygromycin B and 50 µg/mL nourseothricin were sufficient to inhibit mycelial growth of all strains tested (Fig. 1A).

To perform qualitative analysis of stable genomic integration of DNA constructs, we utilized plasmids harboring the coding region of a thermostable red-shifted luciferase that was previously codon-optimized for use in *Paracoccidioides brasiliensis* (*PbLuc*) (27) and expressed under the control of *Paracoccidioides* promoters from the *elongation factor 1-gamma* (*ef1*), *enolase 1* (*eno1*), or *actin* (*act*) genes. These plasmids had been shown to drive functional luciferase expression in both *Aspergillus niger* and *Paracoccidioides* species (Fig. S1A to D) (27).

Protoplasts from *Sporothrix* mycelia were generated by enzymatic digestion of cell wall components using young and actively growing mycelia (25). The fusion of protoplasts and the uptake of the plasmid harboring P*ef1:PbLuc_hph,* P*eno1:PbLuc_hph, or* P*act:PbLuc_hph* was mediated by PEG transformation as previously described for aspergilli (Fig. 1B) (26, 28, 29). This procedure resulted in bioluminescent transformants for all three strains tested (Ss54, Ss126 and Ss469) (Fig. 1C). A selection of bioluminescent transformants was passaged at least 10 times in the absence of the selection marker hygromycin B. The insertion of luciferase into the genome and its expression proved to be stable for at least 3 years, which confirms a stable genomic integration of the reporter construct.

### CRISPR/Cas9 gene editing is an efficient approach for gene disruption in *Sporothrix*

*S. schenckii* and *S. brasiliensis* produce colonies with brownish mycelia when cultivated on solid medium due to the accumulation of melanin pigments formed *via* the DHN-melanin pathway (15, 17, 30). We identified the putative polyketide synthase gene (*pks1*; SPSK_00653 and SPBR_06313) likely to be responsible for the production of 1,3,6,8-tetrahydroxynaphthalene of this DHN-melanin pathway in *Sporothrix* due to its sequence similarity to the *Colletotrichum lagenarium pks1* gene (31) and the *Aspergillus fumigatus* naphthopyrone synthase *pksP* gene (32, 33). We, therefore, targeted the *pks1* for gene deletion using the CRISPR/Cas9 system.

**Fig 2:**
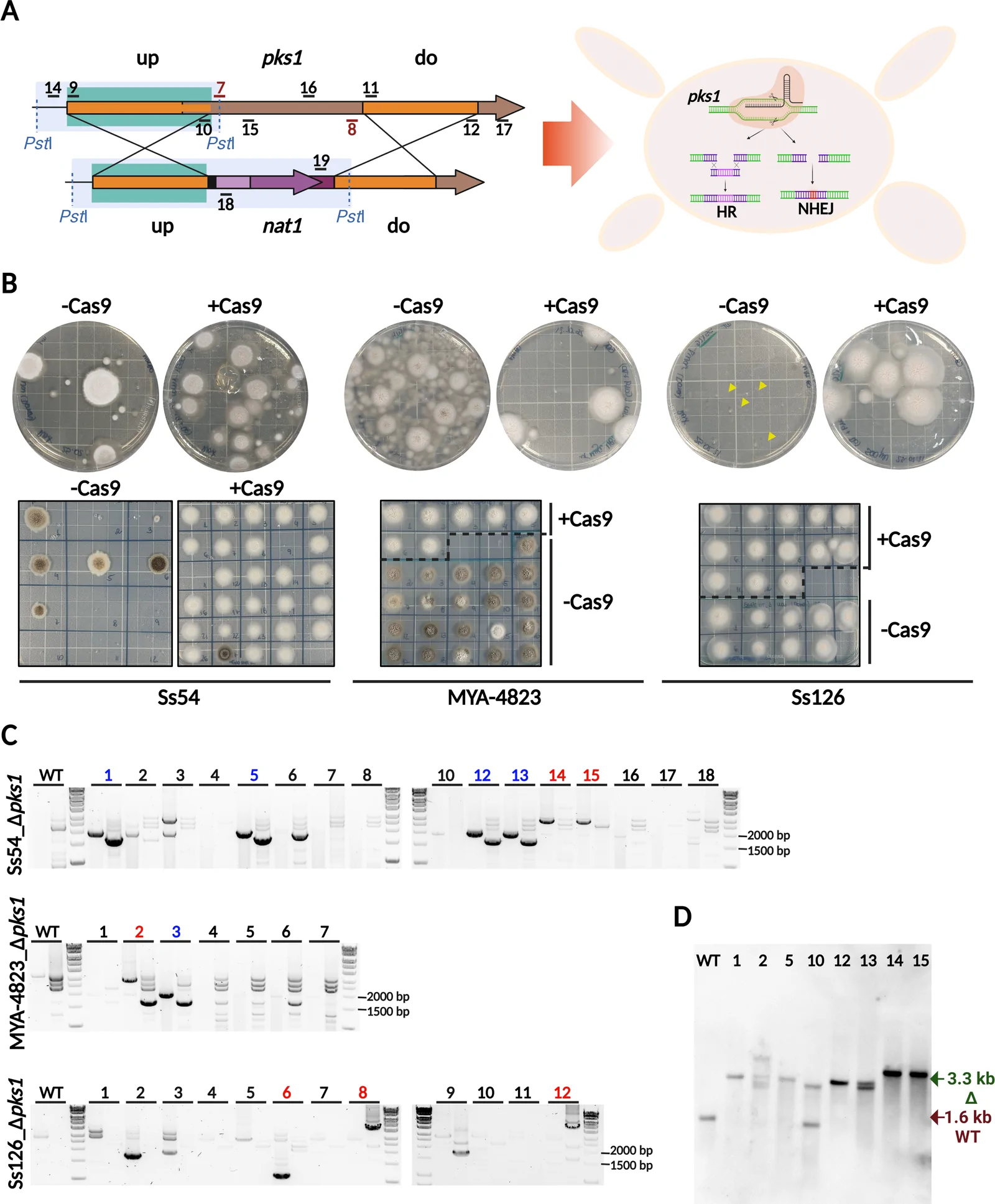
Disruption of the *pks1* gene. (**A**) Schematic representation of the *pks1* deletion construct indicating: the annealing position of oligonucleotides (black) and gRNAs (red) utilized during the gene disruption and transformants selection, the crossover regions predicted for homologous recombination (HR), and the HR and non-homologous end-joining (NHEJ) pathways for repair of DNA double-strand breaks active during gene disruption. The green and blue boxes indicate the probe and restriction sites used for the southern blot analysis, respectively. (**B**) Representative transformation agar plates for *Sporothrix* strains Ss54, MYA-4823, and Ss126 (above) along with subculture of putative *pks1* transformants onto GG10_THI_ containing nourseothricin 80 µg/mL (below). (**C**) PCR analysis for detection of the partial replacement of *pks1* coding region by the deletion cassette containing nourseothricin resistance gene. The insertion of the deletion cassette via homology-directed repair in the upstream flanking region resulted in PCR products of 1,939 and 1,637 bp (samples highlighted in blue) or insertion via non-homologous repair mechanisms (PCR products ≠ 1,939 and 1,637 bp, samples highlighted in red). (**D**) Southern blot analysis of Ss54 transformants that with integration of the deletion cassette on both flanking regions (1, 5, 12, and 13), on the upstream flanking region (14 and 15), or no integration (2 and 10) during the PCR screening. Diagnostic bands of 1.6 kb for wild type (WT) and 3.3 kb for a deletion mutant (Δ) are shown.

Deletion of the *pks1* gene was predicted to eliminate the biosynthesis of DHN-melanin resulting in white mycelia. This loss of mycelial color was used to pre-select for transformants in which the *pks1* gene had been successfully inactivated. Regions of about 850 bp of flanking homology lying upstream and downstream of the Cas9 enzyme cleavage sites in the *Sporothrix pks1* gene were amplified and fused with a nourseothricin (*nat1*) resistance cassette (Fig. 2A; Table S1). Guide RNAs (gRNAs) complexed with Cas9 were used to create the *pks1* gene deletion in Ss126 *S. schenckii*, MYA-4823 and Ss54 *S. brasiliensis* wild-type (WT) strains. Only gRNAs with no predicted off-target effects were used in this study. Two different gRNAs were designed to cut at about 29 and 2,199 bp after the predicted ATG start codon, corresponding to an excision of the first protein domain (acyltransferase) plus part of the second domain (ketosynthase) of the putative polyketide synthase Pks1. The excised *pks1* fragment was replaced by a stop codon sequence plus a *nat1* resistance cassette of approximately 1.7 kb excluding the homology arms.

Transformations were performed with or without Cas9 in parallel to evaluate whether the flanking homologies from the donor DNA were sufficient for targeted gene disruption. During transformation of *S. schenckii* Ss126, the absence of Cas9 resulted in colonies with a smaller size on the transformation agar. However, these transformants showed the expected growth pattern in GG10_THI_ supplemented with nourseothricin (Fig. 2B), indicating that these transformants had incorporated the deletion cassette into the genome.

Interestingly, none of the strains generated brown colonies on the transformation agar, initially suggesting that the *pks1* gene might have been successfully inactivated in the presence or absence of Cas9. However, in some cases, this appeared to derive from the osmotic stabilizer sorbitol in the transformation plates as the transfer of colonies to GG10_THI_ medium lacking sorbitol and supplemented with nourseothricin did result in the development of a brownish color for some Ss54 and MYA-4823 transformants, mostly for colonies generated during the transformation without Cas9 (Fig. 2B). Interestingly, none of the colonies from the *S. schenckii* Ss126 transformation developed a brownish color in GG10_THI_ or potato dextrose agar (PDA), suggesting successful deletion of *pks1*.

To test whether the *pks1* gene had really been disrupted in these transformants, we performed diagnostic PCR analyses on genomic DNA (gDNA). Oligonucleotides flanking the upstream and downstream regions of the excised *pks1* sequence were used to differentiate between the genotypes of WT strains and *pks1-*deleted transformants. We tested 16 Ss54 transformants, 7 MYA-4823 transformants, and 12 Ss126 transformants that were generated using Cas9 (Fig. S2A). We also tested 5 Ss54 colonies, 9 Ss126 colonies, and 13 MYA-4823 colonies that were derived from transformations lacking Cas9 and receiving only donor DNA (Fig. S2B). The WT coding region yielded PCR products of 2,257 and 2,337 bp for the upstream and downstream regions, respectively, whereas *pks1* deletion or recombination was assumed to produce no PCR products. For the tested transformants, approximately 75%, 86%, and 75% of the transformants generated using Cas9 showed the expected disruption of the *pks1* coding sequence in Ss54, MYA-4823, and Ss126, respectively (samples highlighted in purple in Fig. S2A). On the other hand, the *pks1* gene was only disrupted in its downstream region in one Ss54 transformant and one MYA-4823 transformant generated without Cas9 (samples highlighted in green in Fig. S2B).

The transformants were tested further by positional PCR for the integration of the *nat1* deletion cassette into the *pks1* locus (Fig. 2C; Table 1; Fig. S2B). The insertion of the disruption cassette within the *pks1* locus mediated by homology-directed repair resulted in PCR products of 1,939 and 1,637 bp for the upstream and downstream regions, respectively, while an intact WT *pks1* coding region was not expected to result in PCR products. For transformations without Cas9, no insertion of the *nat1* deletion cassette was detected into the *pks1* locus (Fig. S2B; Table 1). In contrast, transformations with Cas9 yielded eight Ss54 transformants, two MYA-4823 transformants, and six Ss126 transformants with insertion of the *nat1* deletion cassette into the *pks1* locus. However, precise *pks1* gene deletion only occurred in four Ss54 transformants and one MYA-4823 transformant (Fig. 2, samples highlighted in blue). Furthermore, at least two Ss54 transformants, one MYA-4823 transformant, and three Ss126 transformants had PCR amplicons with unexpected size, indicating that the integration of the deletion cassette occurred via non-homologous DNA repair mechanisms (samples highlighted in red). It is worth noting that WT and transformants from Ss126 strain did not develop brownish color when cultivated in GG10_THI_, GG10_THI_ supplemented with 2 mM H_2_O_2_ or PDA, making it impossible to distinguish the parental strain from a successful *pks1* gene deletion by the visual screening of transformants. Southern blot analysis was performed on Ss54 transformants to test whether the donor DNA had integrated only at the target locus or ectopically at additional sites (Fig. 2D). The majority of the transformants tested revealed a single integration event. The data indicate that CRISPR/Cas9 promotes effective targeted mutation in *Sporothrix*. As expected, in *S. brasiliensis,* the disruption of the *pks1* locus resulted in white colonies, showing that the gene is required for the brown coloration of mycelia. Unfortunately, a significant proportion of the transformants resulted from non-homologous ectopic integration events, which reduced the number of transformants carrying a true *pks1* gene deletion in *Sporothrix* species.

**TABLE 1:** Estimation of the disruption or deletion of the *pks1* coding region by homologous integration of the *nat1* deletion cassette in *Sporothrix* wild-type and Δ*ku80* strains transformed with or without Cas9.

						pks1 coding region disrupted^*c*^			
Strain	Parental strain background	Cas9	White colonies^*a*^	Brown colonies^*a*^	Nourseothricin^R^, pks1 still present (ectopic integration)^*b*^	Total number transformants with pks1 disrupted	nat1 replaced the pks1 coding sequence (HR)^*h*^	nat1 was inserted at 5' or 3' end of pks1 (non-HR)	nat1 was integrated only in another locus (ctopic integration)^*d*^
Ss54	WT	+	16	0	4/16 (25%)	12/16 (75%)	4/16 (25%)	4/16 (25%)	4/16 (25%)
		−^*i*^	0	5	5/5 (100%)	0/5 (0%)	0/5 (0%)	0/5 (0%)	0/5 (0%)
	Δ*ku80*	+	13	0	0/13 (0%)	13/13 (100%)	11/13 (85%)	2/13 (15%)	0/13 (0%)
		−	0	9^*e*^	ND^*g*^	ND	ND	ND	ND
MYA-4823	WT	+	7	0	1/7 (14.3%)	6/7 (85.7%)	1/7 (14.3%)	1/7 (14.3%)	4/7 (57.1%)
		−	0	13	13/13 (100%)	0/13 (0%)	0/13 (0%)	0/13 (0%)	0/13 (0%)
	Δ*ku80*	+	13	0	0/13 (0%)	13/13 (100%)	13/13 (100%)	0/13 (0%)	0/13 (0%)
		−	0	16^*e*^	ND	ND	ND	ND	ND
Ss126	WT	+	12	0	3/12 (25%)^*f*^	9/12 (75%)	0/12 (0%)	6/12 (50%)^*f*^	3/12 (25%)
		−	9	0	9/9 (100%)	0/9 (0%)	0/9 (0%)	0/9 (0%)	0/9 (0%)

^*a*^ Color of the colonies tested in diagnostic PCRs.

^*b*^ These transformants were nourseothricin resistant, but the *pks1* locus was present (PCR products for 5' and/or 3' coding region were detected).

^*c*^ PCR products for the *pks1-*coding sequence were not detected.

^*d*^ PCR showed that *pks1-*coding sequence was deleted from the genome. However, *nat1* deletion cassette was not detected at the *pks1 locus*, indicating ectopic integration of *nat1.*

^*e*^ Colonies not tested for coding region disruption or homologous recombination of *nat1* deletion cassette.

^*f*^ Colony 8: PCR products were detected for 5'-end of the *pks1-*coding region and 3'-end of the *nat1* deletion cassette, suggesting aberrant insertion of *nat1* at the *pks1* locus. For the calculations, this colony was added to this group: “*nat1* was inserted at 5' or 3' end of *pks1.*”

^*g*^ ND, not determined.

^*h*^ HR, homologous recombination.

^*i*^ -, transformations without Cas9.

### Enhancing homologous recombination in *S. brasiliensis*

**Fig 3:**
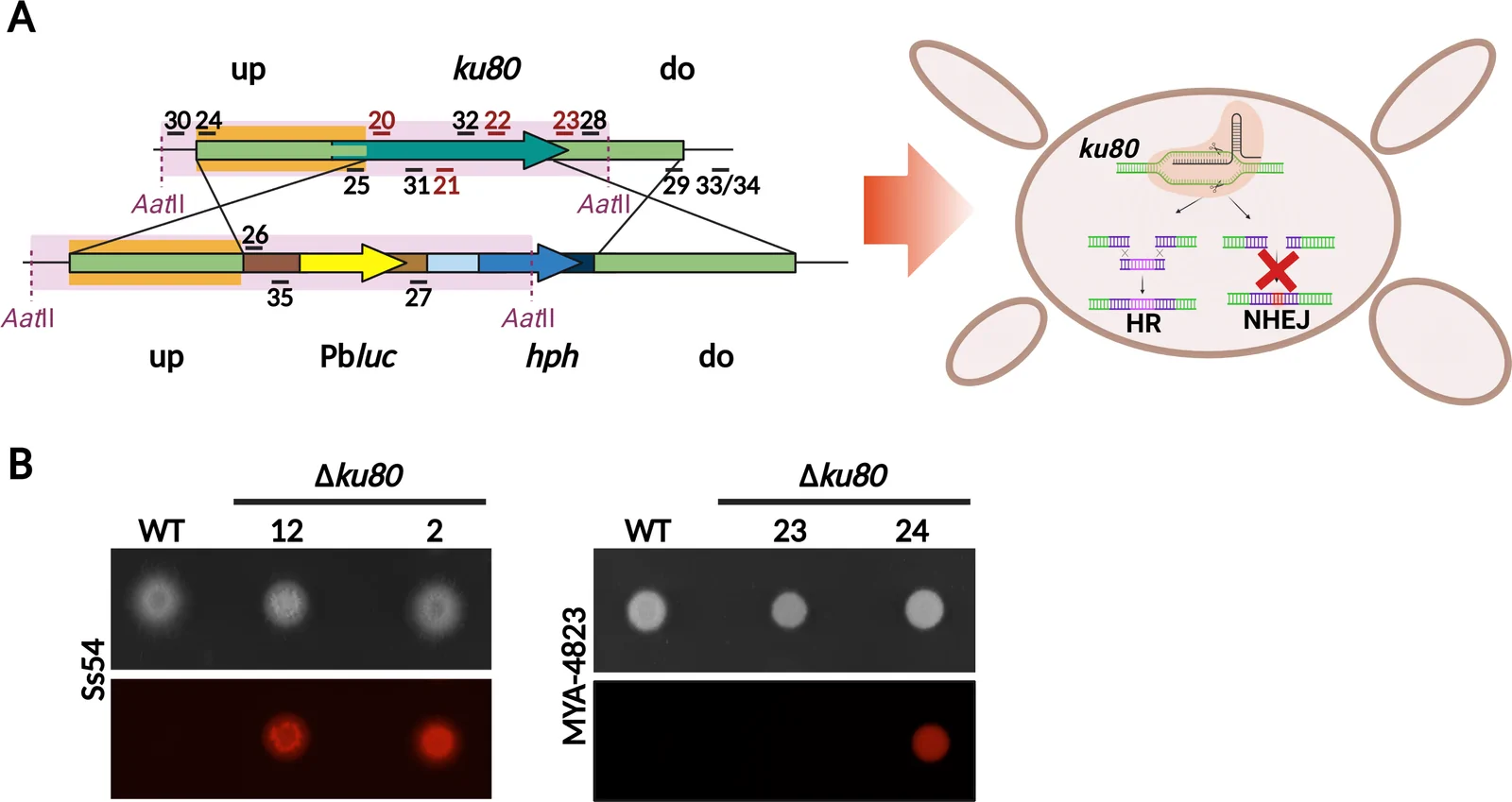
Generation of non-homologous end joining deficient strains of *Sporothrix brasiliensis*. (**A**) Schematic representation of *ku80* deletion cassette containing the *hph* resistance gene, the annealing position of oligonucleotides (black), and gRNAs (red), as well as the schematic representation of the resultant strain defective in NHEJ pathway. The orange and pink boxes indicate the probe and restriction sites used for the southern blot analysis, respectively. (**B**) Bioluminescent imaging of two transformants of Ss54 and MYA-4823 containing in locus integration of the *ku80* deletion cassette inoculated onto GG10_THI_ containing d-luciferin (0.2 mM). Brightfield (upper panels) and luminescence (bottom panels) were recorded after 5 days of incubation at 25°C.

Our results showed that the *pks1* gene from *Sporothrix* can be successfully disrupted if Cas9-mediated transformation is applied. However, double crossover events resulting in a gene deletion, rather than disruption, by Cas9-mediated DNA excision were less frequently observed. Therefore, we aimed to improve gene targeting by deleting the *ku80* (SPBR_02356 and SPSK_07043) gene. The absence of this gene should favor a homologous recombination-mediated repair during the transformation process (34). To achieve this, we transformed both strains of *S. brasiliensis* with a deletion cassette containing the hygromycin B resistance gene (*hph*), followed by the *PbLuc* luciferase expression construct, and flanking homology arms to the upstream and downstream regions of the *ku80* gene (designed to delete the entire *ku80* coding sequence) (Fig. 3A; Table S1). As Pef1:PbLuc_hph, Peno1:PbLuc_hph, or Pact:PbLuc_hph were able to generate bioluminescent transformants in *Sporothrix,* the *Paracoccidioides ef1* promoter-*PbLuc* marker was chosen due to the highest bioluminescence levels observed in single integration mutants of *Aspergillus niger* (Fig. S1A to D) (27). Putative hygromycin B resistant *ku80* ^−^ transformants were tested for the presence of the resistance/reporter construct by diagnostic PCR. Of the 72 transformants tested for each strain, three Ss54 colonies and two MYA-4823 colonies ultimately showed a single-copy integration of the donor DNA at the expected site when tested by Southern blot analysis (Fig. S3). Most of these transformants also displayed the expected bioluminescence from luciferase activity (Fig. 3B). Strains with homology-directed repair in either the upstream or downstream regions were also identified in addition to the transformants in which the *ku80* gene had been successfully deleted.

### *ku80* gene deletion enhances targeted DNA integration in *S. brasiliensis*

To test whether the *ku80* deletion enhances targeted integration of the donor DNA during transformation, we repeated the *pks1* deletion using the *S. brasiliensis* strains Ss54_Δ*ku80*_12 and MYA-4823_Δ*ku80*_24. In the presence of Cas9, we obtained 25 transformants from the Ss54_Δ*ku80* transformation, 13 of which produced white colonies in the first streak on GG10_THI_ supplemented with nourseothricin. The remaining 12 brown colonies did not survive the subsequent passages for isolation of conidia from the transformants. This indicated that in these putative transformants the gene deletion construct had not been stably integrated into the genome. This left only white colonies as stable transformants from this transformation. In the MYA-4823_Δ*ku80* transformation, 32 colonies were recovered with 25 forming a white colony and seven showing a brown phenotype. The *pks1* transformations lacking Cas9 only generated brown colonies when MYA-4823_Δ*ku80* or Ss54_Δ*ku80* were utilized as parental strains.

**Fig 4:**
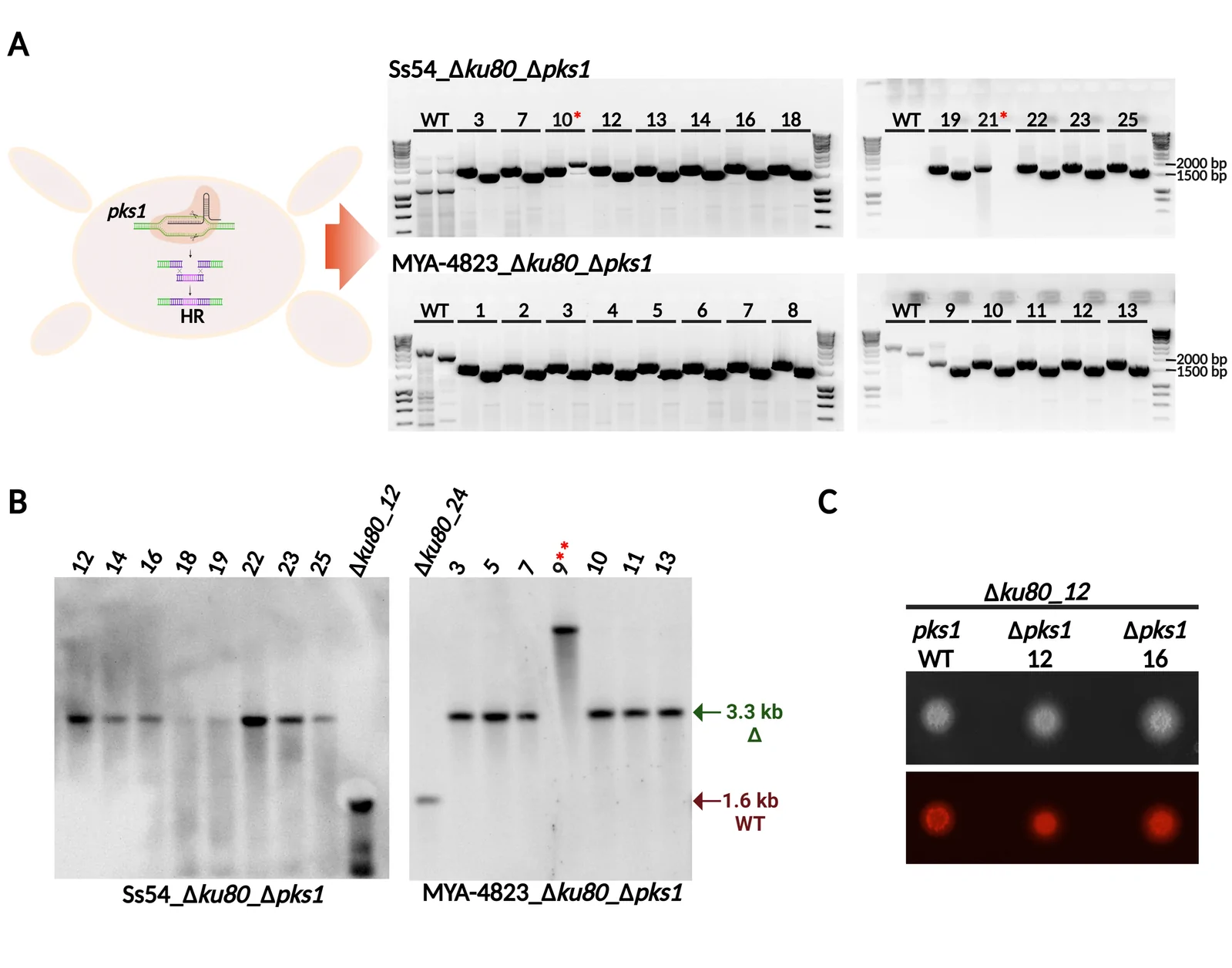
Enhanced homologous recombination by Δ*ku80* strains. (**A**) PCR analysis of putative Δ*ku80_* Δ*pks1* transformants to confirm the disruption of *pks1* gene and in locus integration of the nourseothricin deletion cassette using oligonucleotides for the deletion construct. For each transformant, the left-hand lane represents PCR diagnosis of the upstream integration site, and the right-hand lane represents the diagnosis of the downstream integration site: (*) samples lacking one of the PCR products or containing fragments of unexpected size. (**B**) Southern blot analysis of randomly selected Δ*ku80*_Δ*pks1* transformants from *S. brasiliensis* strains. Diagnostic bands of 1.6 kb for wild type (WT) and 3.3 kb for a deletion mutant (Δ) are highlighted: (**) samples containing a band of unexpected size. (**C**) Bioluminescence imaging of two Ss54_Δ*ku80*_Δ*pks1* transformants and their parental strain Ss54_Δ*ku80*_12; 6 × 10^5^ conidia were spotted onto GG10_THI_ plates containing d-luciferin (0.2 mM). Brightfield (upper panel) and luminescence (bottom panel) were recorded after 5 days of incubation.

For the Cas9-mediated transformation, diagnostic PCR analysis was performed on 13 white transformants from each strain to evaluate the effect of *ku80* gene deletion on the accuracy of gene targeting. We observed that accurate targeted deletion of *pks1* had taken place in 100% of the MYA-4823_Δ*ku80* transformants and 85% of the Ss54_Δ*ku80* transformants (Fig. 4A; Table 1). Furthermore, Southern blot analysis of seven MYA-4823_Δ*ku80*_Δ*pks1* transformants and eight Ss54_Δ*ku80*_Δ*pks1* transformants confirmed a single-copy integration of the deletion cassette in all but one of the MYA-4823_Δ*ku80*_Δ*pks1* transformants analyzed (Fig. 4B).

To verify the stability of integration of the exogenous DNA across the transformations, conidia from two of the Ss54_Δ*ku80*_Δ*pks1* transformants and their parental strains were spotted on GG10_THI_ supplemented with d-luciferin. Bioluminescence imaging confirmed the stable expression of the luciferase in the Ss54_Δ*ku80*_Δ*pks1* transformants after this second round of transformation and gene deletion (Fig. 4C). We concluded that the deletion of *ku80* promotes the precise integration of the donor DNA during Cas9-mediated transformation, facilitating the screening process for true gene deletion mutants in *S. brasiliensis*. Furthermore, these results indicate that, in contrast to other fungi studied so far (34 – 36), a lack of KU80 alone is not sufficient to promote homologous gene replacement in *S. brasiliensis* but requires the induction of DNA-strand breaks by the Cas9.

### *S. brasiliensis* Pks1 mediates oxidative stress protection

**Fig 5:**
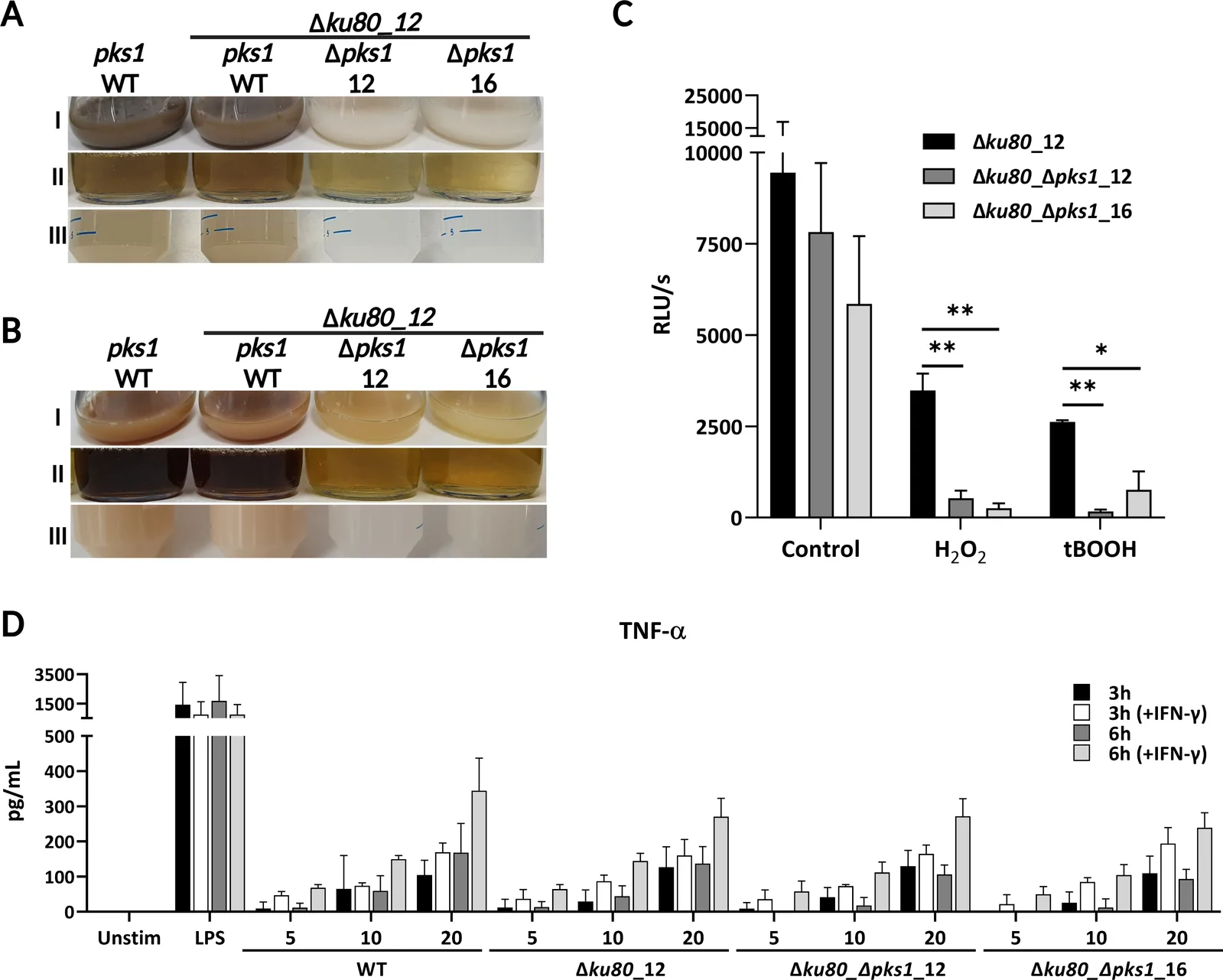
Phenotypic characterization of *Sporothrix* deletion strains. (**A**) The mycelial phase of Ss54 WT, Ss54_Δ*ku80*_12, Ss54_Δ*ku80*_Δ*pks1*_12, and Ss54_Δ*ku80*_Δ*pks1*_16 strains was cultivated in Sabouraud dextrose at 25°C and 150 rpm for 19 days. (**B**) Yeast phase of the same strains cultivated in YPD at 37°C and 200 rpm for 7 days. For (**A and B**), (**I**) conidia or yeast cultures; (II) culture supernatants; and (III) suspensions of washed conidia or yeast cells. (**C**) Bioluminescence assay of yeast cells cultured in RPMI containing 0.4 mM d-luciferin and exposed to 20 mM hydrogen peroxide (H_2_O_2_) or 8 mM tert-butylhydroperoxide (tBOOH) for 8 h. Each assay was performed in technical triplicate and repeated twice, and the statistical differences were calculated by mean values using one-way ANOVA. *P < 0.05; **P < 0.01. (**D**) TNF-α levels after 3 or 6 h of co-incubation of bone marrow-derived macrophages (BMDMs) with opsonized yeast cells from Ss54 WT and *pks1* deletion strains. BMDMs were either primed or not primed with IFN-γ and were co-incubated with yeast cells at an MOI of 5, 10, or 20.

We tested whether Pks1-dependent melanin production, most likely producing a DHN-melanin, is responsible for the brown pigmentation of yeasts and mycelia during the long-term cultivation in liquid cultures. Sabouraud and yeast peptone dextrose (YPD) media were inoculated with conidia from two independent Ss54_Δ*ku80*_Δ*pks1* mutants and their parental Δ*ku80* and wild-type strains. After 19 days of growth at 25°C, the mycelia from Ss54 WT and Δ*ku80* strains, but not from the Δ*ku80*_Δ*pks1* strains, developed a strong brown pigmentation (Fig. 5A). Similarly, Ss54 WT and Δ*ku80* yeasts cultivated in YPD at 37°C for at least 7 days showed a darker pigmentation when compared with the Δ*ku80*_Δ*pks1* strains (Fig. 5B).

The production of melanin is a virulence determinant due to the ability of melanin to scavenge free radicals. This contributes to resistance of fungal pathogens to host-pathogen interactions, as well as to environmental stresses (37). A previous study had suggested that inhibition of the DHN-melanin pathway results in impaired survival of *S. schenckii* to oxidative stress as induced by hydrogen peroxide (H_2_O_2_) (15). Therefore, to test whether *pks1* deficient strains are more sensitive to oxidative stress, we measured the light emission from the luciferase of live cells after exposure to H_2_O_2_ or tert-butylhydroperoxide (tBOOH). The bioluminescence emitted by firefly luciferases is ATP-dependent and can, thus, be used as a viability marker (38). In comparison to the parental strain, exposure to 20 mM H_2_O_2_ significantly decreased the intensity of the bioluminescence generated by both Ss54_Δ*ku80*_Δ*pks1* mutants tested (Fig. 5C). Similarly, the bioluminescence signal intensity was significantly higher from the parental strain Ss54_Δ*ku80* than from the Δ*pks1* mutants after exposure to 8 mM tBOOH. This suggests that the presence of a functional *pks1* gene confers protection against the oxidative stresses imposed by tBOOH and H_2_O_2_.

To analyze whether Pks1 influences fungus-host interactions, bone marrow-derived macrophages (BMDMs) were primed or not with interferon-γ (IFN-γ) and co-incubated with opsonized yeasts of Ss54 WT, Ss54_Δ*ku80*_12, and two Ss54_Δ*ku80*_Δ*pks1* mutants for 3 and 6 h at a multiplicity of infection (MOI) of 5, 10, and 20. Tumor necrosis factor alpha (TNF-α) was measured from the supernatants. TNF-α secretion by BMDMs was proportionate to the MOI and priming of the cells with IFN-γ resulted in a slight but non-significant increase of TNF-α secretion. Neither deletion of *ku80* nor *ku80* plus *pks1* deletion resulted in a modified secretion of TNF-α by BMDMs compared to the response induced by Ss54 WT yeasts (Fig. 5D). Therefore, we conclude that inactivation of *pks1* significantly affects sensitivity of *Sporothrix* cells to reactive oxygen species but does not alter the release of pro-inflammatory cytokines from macrophages, at least under the conditions examined.

## DISCUSSION

Genetic engineering is a key tool for dissecting the molecular mechanisms that underlie virulence and for facilitating the search for more efficient antimicrobial therapies. *Sporothrix* species have been considered challenging for genetic manipulation (24), and only limited tools are available for applying reverse genetics to resolve molecular mechanisms critical for sporotrichosis development (15, 19 – 23). RNA interference-mediated gene silencing using *Agrobacterium*-mediated transformation has been used for studying the Golgi α-1,6-mannosyltransferase (*Och1*) and protein rhamnosylation (*rmlD*) genes in *S. schenckii* (22, 23). However, so far, attempts to generate platform strains to facilitate the screening of transformants and to monitor the effects of gene deletion or overexpression in *Sporothrix* as model organism have not been reported.

In this study, we have successfully established an efficient protocol for the accurate integration of exogenous DNA into the genomes of *S. brasiliensis*, *S. chilensis,* and *S. schenckii*. We applied PEG-mediated fusion of protoplasts that were generated from the mycelial phase to stably incorporate a plasmid containing *hph* and *PbLuc* genes into the *Sporothrix* genome. Light emission from the luciferase reporter was still detectable after a vegetative sub-cultivation period of 3 years, which is consistent with the stable integration of genes into the genomes of *Sporothrix* species. In addition, the functional expression of a red-shifted luciferase enables real-time monitoring of *in vivo* of disease progression as shown for other fungal species such as *Aspergillus fumigatus*, *Aspergillus terreus*, *Candida albicans,* and *Cryptococcus neoformans* (38 – 46). Our developments on genetic tools to study *Sporothrix* species complex been reinforced by a parallel study that also describes approaches for heterologous gene expression in *S. brasiliensis* (47).

The Cas9-mediated gene deletion methodology we developed evolved from many distinct unsuccessful attempts. Protocols described for yeast and mold transformation, such as electroporation, lithium acetate, and heat-shock yeast transformation (25, 48, 49) did not generate *Sporothrix* transformants in our hands. In PEG-mediated protoplast transformation, deletion cassettes with 800–1,000 bp of homology did not yield deletions in distinct target genes but were integrated ectopically. For targeted gene disruption and deletion, previous studies have reported that flanking homologies ranging in length from 30 to 2,000 bp can yield efficient homology-directed repair after DNA double-strand breaks induced by Cas9 in aspergilli, *Rhizopus microspores* and *C. neoformans* (50 – 52). Therefore, based on the protocols used for gene deletion in *Magnaporthe oryzae* and *Aspergillus* species (29, 53), we developed a CRISPR/Cas9-based protocol for gene disruption in *S. brasiliensis* and *S. schenckii*. By exploiting the putative 1,3,6,8-tetrahydroxynaphtalene synthase gene *pks1* as a visual marker for successful gene disruption/deletion and resistance to nourseothricin as transformation marker, we confirmed that DNA double-strand breaks were successfully induced by Cas9 to generate mutants deficient for DHN-melanin-like pigments. However, even though Cas9 cut precisely at the expected *pks1* Cas9 sites*,* homologous recombination at both flanks was observed at low frequency in wild-type *Sporothrix* backgrounds. Unlike some other fungal species (34 – 36), this suggested a low rate of homology-directed DNA repair in *S. brasiliensis* and *S. schenckii* during the transformation process.

The repair of DNA double-strand breaks is assumed to occur through two main pathways: homologous recombination and non-homologous end joining repair (NHEJ). The NHEJ is the most active pathway for DNA repair in filamentous fungi, which often reduces the efficacy of in locus DNA integration during the transformation process (54). The Ku70/Ku80 heterodimer participates in the initial step of DNA double-strand repair by the NHEJ pathway. For this reason, deletion of either *ku70* or *ku80* encoding genes has been used successfully as a strategy to improve homologous recombination in a diverse range of fungal species (29, 35, 36, 39). Interestingly, while *S. brasiliensis* transformation in the absence of Cas9 did not result in *ku80* deletion mutants, the simultaneous addition of Cas9 to the transformation procedure generated improved the homologous recombination to an index greater than 80%. Importantly, the Δ*ku80* transformants preserved the expected wild-type morphology as well as their thermo-dimorphism and no obvious phenotypic changes were observed during subcultivation.

In terms of the deletion of the *pks1* gene, we observed a change in colony and medium coloration for *pks1* mutants compared to the respective parental wild-type strain, which was consistent with a lack of DHN-melanin production. An increased melanin production is observed during responses to cellular stresses such as starvation, oxidative and nitrosative stresses (55 – 57). Accordingly, deletion of enzymes related to melanin biosynthesis results in attenuated fungal virulence (55 – 60). Previous studies have shown that *S. schenckii* and *S. globosa* UV-induced mutants deficient for DHN-melanin production are more susceptible to oxidative/nitrosative stresses and induce higher levels of reactive oxygen species by human monocytes and murine macrophages (15, 61). Therefore, the sensitivity of *pks1* mutants to H_2_O_2_ and tBOOH observed in this study is consistent with the view that pigments like DHN-melanin protect *Sporothrix* against oxidative stress. Most importantly, our targeted deletion of *pks1* provides a proof of concept for future investigations of point-mutations, insertions, deletions, and promoter replacements, all of which will greatly improve the tools for understanding the virulence mechanisms of *Sporothrix* species.

In summary, our results show that PEG- and CRISPR/Cas9-mediated transformation of protoplasts provides a precise and efficient tool for the genetic manipulation of pathogenic *Sporothrix* species. Thereby, efficient gene targeting by homologous recombination is remarkably enhanced in strains deficient in NHEJ repair. The development of reliable and reproducible tools for genetic engineering of *Sporothrix* opens powerful experimental avenues to improve the understanding of mechanisms underlying the development of sporotrichosis. This is particularly important given the rapid geographic expansion and upsurge of atypical and more severe forms of this debilitating fungal disease.

## MATERIALS AND METHODS

### Strains and growth conditions

*Sporothrix brasiliensis* Ss54, *S. schenckii* Ss126, and *S. chilensis* Ss469 strains were kindly provided by Professor Anderson Messias Rodrigues (Federal University of São Paulo), and the *S. brasiliensis* MYA-4823 strain was acquired from ATCC.

For harvesting conidia, strains were cultivated for 7 days in Sabouraud liquid medium (Sigma) pH 4.5 at 25°C and 150 rpm. Cultures were filtered through Miracloth (Merck) filter gauze, supernatants centrifuged at 3,220 × *g* for 10 min, and the conidia were washed twice in phosphate-buffered saline (PBS; Gibco). Fungal transformants were plated on GG10 medium (26, 62) containing 3 µg/mL thiamine (Sigma) (GG10_THI_) supplemented with 1.2 M sorbitol (Sigma), 80 µg/mL of nourseothricin (nat, Jena Biosciences), or 180 µg/mL of hygromycin B (Thermo) when necessary. Yeast cells were grown at 37°C in yeast peptone dextrose (1% yeast extract-Difco, 2% bacteriological peptone-Oxoid, 2% glucose-Fisher) pH 7.8. All liquid cultures were incubated at 200 rpm on a rotary shaker. For solid YPD medium, 2% (wt/vol) of agar was added prior to sterilization. For macrophage stimulation and oxidative stress assay, conidia suspensions were inoculated in RPMI medium (Sigma) supplemented with 2% (wt/vol) glucose and non-essential amino acids (Gibco), pH 7.2 (abbreviated as RPMI 2% glucose hereafter), and incubated at 37°C and 200 rpm for 6 (cytokine production) and 7 days (oxidative stress assay).

### Kits and reagents

DNA amplifications for cloning and transformant screening were performed with Phusion Green High-Fidelity DNA Polymerase (Thermo) or Phire Green Hot Start II DNA Polymerase (Thermo), respectively. For cloning, the PCR products and digested plasmids were run on 1% or 0.8% agarose gels, and gel elution of DNA fragments was performed using Zymoclean Gel DNA Recovery Kit (Zymo). The *in vitro* assembly of PCR products was performed by using the In-Fusion HD cloning kit (Takara). The assembled plasmids were amplified by transformation of Stellar Competent Cells (Takara). Plasmids were isolated from overnight cultures grown at 37°C in LB medium containing 100 µg/mL Ampicillin using the NucleoSpin Plasmid isolation kit (Macherey-Nagel). The selection markers for fungal transformation were cloned into digested plasmids by using the Rapid DNA Ligation Kit (Thermo). The guide RNAs (gRNAs) were generated by EnGen sgRNA Synthesis Kit (NEB) according to the manufacturer’s protocol. The synthesized gRNA was purified by RNA Clean & Concentrator (Zymo) and assembled to the EnGen Spy Cas9 NLS (NEB) directly prior to the fungal transformation. Oligonucleotides utilized in this study are described in the Table S1.

### Transformation constructs

To establish the transformation protocol for *Sporothrix*, we first tested plasmids that were previously used in *Aspergillus niger* and/or *Paracoccidoides* transformations (27). We also generated the P*ef1:Pbluc_OPT_red_*:T*eno1*_URABlaster_pUC19 by amplification and assembly of the synthetic gene *Pbluc_OPT_red_* (GenBank: MT978127), as well as the *elongation factor 1-gamma* (*ef1,* PAAG_03556) promoter and *enolase 1* (*eno1,* PAAG_11169) terminator from Pb01 gDNA, using the oligonucleotides from 1 to 6. To replace the URABlaster selection marker, P*act:Pbluc_OPT_red_*:T*eno1*_URABlaster_pUC19, P*eno1:Pbluc*:T*eno1*_URABlaster_pUC19 (27), and P*ef1:Pbluc_OPT_red_*:T*eno1*_URABlaster_pUC19 (this study) were digested with *Not*I (Thermo), treated with Alkaline Phosphatase (AP; Thermo), gel purified, and assembled with a *Not*I-restricted hygromycin B (*hph*) resistance cassette.

The gene selected for the assembly of the *pks1* (SPSK_00653; SPBR_06313) deletion cassette was identified from genome searches against the *C. lagenarium pks1* gene (31) and the *A. fumigatus* naphthopyrone synthase *pksP* gene (32, 33). A sequence of 892 bp upstream of *pks1* gene including the first 17 bp of the coding region was amplified by PCR using the oligonucleotides 9 and 10 and MYA-4823 gDNA as template. The 862 bp downstream region of the *pks1* deletion construct was designed starting from the 2,233 bp (*S. brasiliensis*) or 2,229 bp (*S. schenckii*) of *pks1* using oligonucleotides 11 and 12. In the reverse oligonucleotide for the upstream flanking region, a stop codon sequence was included as well as a *Not*I restriction site for cloning of the selection marker. The upstream and downstream flanking fragments of the *pks1* deletion construct were assembled by *in vitro* recombination into a *Sma*I-restricted pUC19 plasmid (Thermo). *Escherichia coli* transformants containing the correctly assembled plasmids were identified by colony PCR using oligonucleotides 12 and 13. The isolated Δ*pks1*_pUC19 plasmids were digested with *Not*I and dephosphorylated by AP. The linearized plasmids were ligated with a *NotI*-digested *nat1* resistance cassette. The correct assembly was confirmed by colony PCR using the oligonucleotides 12 and 19. For *Sporothrix* transformation, the deletion cassette Δ*pks1::nat1* was excised from pUC19 by digestion with *Sma*I. The two gRNAs for *pks1* deletion were synthetized using oligonucleotides 7 and 8.

The ATP-dependent DNA helicase 2 subunit 2 (*ku80*) from *Sporothrix* species (SPBR_02356 and SPSK_07043) were identified by Blast search using the *Aspergillus fumigatus akuB* sequence (AFUA_2G02620) as a template at NCBI or EnsemblFungi websites (https://www.ncbi.nlm.nih.gov/, https://fungi.ensembl.org/index.html). The upstream region of the *ku80* deletion construct (774 bp) including 411 bp of the coding region as well as the downstream region (792 bp) was amplified from Ss126 gDNA using oligonucleotides 24 and 25 or 28 and 29, respectively. The P*ef1:Pbluc_OPT_red_*:T*eno1* fragment was amplified from the plasmid described above using oligonucleotides 26 and 27. The three PCR products were assembled in a *Sma*I digested pUC19 plasmid by *in vitro* recombination. The Δ*ku80::Pbluc_OPT_red__*pUC19 plasmid was linearized by *Not*I, dephosphorylated, and assembled with the *Not*I-restricted *hph* selection marker. For *Sporothrix* transformation, the deletion cassette Δ*ku80::Pbluc_OPT_red_* _*hph* was released from the pUC19 backbone by *Sma*I-restriction and gel purified. The four gRNAs utilized for the *ku80* deletion were synthetized using oligonucleotides 20, 21, 22, and 23. All gRNAs were designed using the Eukaryotic Pathogen CRISPR guide RNA/DNA Design Tool (http://grna.ctegd.uga.edu/) and off-target analyses were performed on Ensembl Fungi website (http://fungi.ensembl.org/index.html) according to the parameters previously described (63).

### Transformation

*Sporothrix* conidia suspensions were inoculated in 50 mL of Sabouraud pH 4.5 and grown for 48–72 h at 25°C and 150 rpm to obtain actively growing mycelium that has not reached the stationary growth phase (which is generally reached after 5–7 days under the selected conditions). Protoplasts were prepared as described previously for *Aspergillus* species (29) with some modifications. The mycelium was collected and washed with sterile tap water over sterile Miracloth filter gauze and transferred into 30 mL of 90 mM citrate/phosphate buffer pH 7.3 supplemented with 10 mM 1,4-dithiothreitol (Thermo) and incubated for 1 h at 25°C. The mycelium was collected and washed with citrate/phosphate buffer over sterile Miracloth and transferred to 20 mL of protoplasting solution (0.6M (NH_4_)_2_SO_4_, 50 mM maleic acid buffer pH 5.5) containing 100 mg Yatalase (Takara), and 100 mg Lysing Enzymes from *Trichoderma harzianum* (Lot: SBLJ0553V; Sigma). Samples were incubated at 25°C and 70 rpm, and the release of protoplasts was monitored under the microscope for up to 90 min. Protoplasts were separated from the mycelium by filtration over sterile Miracloth, pelleted at 3,220 × *g* for 8 min at 4°C, washed once in 20 mL of washing solution (0.6 M KCl, 0.1 M Tris/HCl, pH 7.0), followed by resuspension in 10 mL of solution A (50 mM CaCl_2_, 0.6 M KCl, 0.1 M Tris/HCl pH 7.5). The number of protoplasts was evaluated using a Neubauer counting chamber, and the protoplasts were finally resuspended in solution A to a concentration of about 5 × 10^6^ to 2 × 10^7^ protoplast/mL and 100 µL of this suspension was used in the transformation procedure.

The uptake of donor DNA and Cas9 by protoplasts was adapted from protocols previously described for *Magnaporthe oryzae* and *Aspergillus species* (29, 38, 53, 64). The Cas9/gRNA complex was generated as described by the manufacturer’s protocol (NEB). For *pks1* deletion, a total of 80 pmol of each synthetized gRNA was mixed with 40 pmol of Cas9, in two different microtubes, and incubated for 10 min at 25°C before adding the mixture to the protoplasts. One microgram of deletion cassette was added to a 2-mL microtube together with the two Cas9/gRNA complexes. In transformations performed only in the presence of the donor DNA, 1.5 µg of the deletion cassette was added to each microtube. For targeting the *ku80* gene, the gene deletion was performed with 9.5 pmol Cas9 per 19 pmol gRNA for each of the gRNAs utilized. One microgram or 1.5 µg of deletion cassette was added to 2 mL microtubes with or without Cas9/gRNA complexes, respectively. The 100 µL of the protoplast suspension was mixed with 25 µL of PEG solution (25% PEG 8000, 50 mM CaCl_2_, 10 mM Tris/HCl, pH 7.5) in a 2-mL microtube containing Cas9/gRNA complexes and the donor DNA or only the latter. The microtube was incubated for 25 min at room temperature and another 500 µL of PEG solution was added. After 5 min, 1 mL of solution A was added and the protoplast suspension and transferred to 5 mL of liquid Sabouraud medium supplemented with 1.2 M sorbitol, pH 4.5. For regeneration, the protoplasts were incubated overnight at 25°C at 100 rpm, pelleted at 3,220 × *g* for 8 min at room temperature, washed twice in 5 mL of solution A, and resuspended in 1.6 mL of solution A. Aliquots of 400, 500, and 700 µL of the regenerated protoplast suspension were plated on individual agar plates of GG10_THI_ supplemented with 1.2 M Sorbitol and 80 µg/mL of nourseothricin or 180 µg/mL of hygromycin B.

### Molecular analysis of transformants

After three passages in GG10_THI_ supplemented with nourseothricin or hygromycin B, conidia from selected transformants were transferred to a 48-well plate containing 500 µL of YPD and incubated for 5 days at 37°C and 180 rpm on a rotary shaker incubator. The cells were harvested in screw-cap tubes containing Lysing Matrix Y (MP Biomedicals) and pelleted at 10,000 × *g* for 10 min at 4°C. The supernatant was discarded, 500 µL of extraction buffer (100 mM Tris/HCl, 50 mM EDTA, 500 mM NaCl, 10 mM β-mercaptoethanol, 1% SDS, 20 µg RNAse A) was added, and the cells were disrupted in a FastPrep machine (MP Biomedicals) in three rounds of 30 s at 6,000 rpm. Alternatively, 400 µL Buffer AP1 and 40 µg RNase A were added to the pelleted yeasts and the DNA extraction performed according to the instructions from the DNeasy Plant Mini Kit (Qiagen). For DNA extractions used in Southern blot analyses, protoplasts were prepared as described in the transformation procedure, but 1.3 g of VinoTaste Pro (Novozyme) in osmotic solution (0.6 M KCl, 10 mM phosphate buffer pH 5.7) was utilized instead of the yatalase enzyme. After overnight incubation at 25°C and 70 rpm, the protoplasts were separated from the mycelium by filtration over Miracloth, pelleted at 3,220 × *g* for 8 min at 4°C, resuspended in 500 µL of extraction buffer, and transferred to 2 mL microtubes. The subsequent purification of gDNA from disrupted yeast cells and from protoplasts was extracted as described by Dellaporta et al. (65). The gDNA from protoplasts preparations was purified from 0.8% agarose gels using the Large Fragment DNA Recovery Kit (Zymo).

A pre-screening of *pks1* deletion mutants was performed by PCR to verify the absence of the respective coding region (oligonucleotides 14 and 15 or 16 and 17) and the in locus insertion of the deletion constructs (oligonucleotides 14 and 18 or 17 and 19). The PCRs on the downstream region were performed in the presence of 6% DMSO to favor the amplification of this GC-enriched region. The *ku80* deletion strains were similarly screened for the excision of the entire coding region (oligonucleotides 30 and 31 or 32 and 33/34) and the in locus insertion of *luciferase* and *hph* genes (oligonucleotides 30 and 35, 26 and 33/34, or 30 and 33/34). In addition, selected Δ*ku80* and Δ*ku80*_Δ*pks1* strains were plated on Sabouraud and YPD agar plates supplemented with 0.4 mM of d-luciferin potassium salt (Promega) to confirm the expression of a functional luciferase through bioluminescence imaging using ChemiDoc MP (Bio-Rad).

### Southern blot analysis

To confirm the homologous integration of the deletion construct into the genome, gel-purified gDNA from potential Δ*pks1* and Δ*ku80_*Δ*pks1* transformants and their parental strains were digested overnight by *Pst*I (Thermo). gDNA of Δ*ku80* strains was digested overnight with *Aat*II (Thermo). Digested gDNA was loaded on a 0.8% agarose gel, separated by electrophoresis, and transferred to a nylon membrane Hybond *N* ^+^ (GE Healthcare). The probes for the upstream regions of *pks1* (oligonucleotides 9 and 10) and *ku80* (oligonucleotides 24 and 25) deletion constructs were synthetized using PCR DIG DNA Labelling Mix (Roche) and Taq DNA Polymerase (NEB). Probes were hybridized overnight in DIG Easy Hyb (Roche) at 42°C and detection was performed by chemiluminescence using anti-Digoxigenin-AP Fab fragments (Roche) and CDP-Star chemiluminescent substrate (Roche) in a ChemiDoc MP (Bio-Rad).

### Oxidative stress assay

Yeast cells of selected strains were cultivated in RPMI 2% glucose, pelleted at 3,220 × *g* for 10 min at 4°C, washed twice in PBS, and resuspended at a concentration of 1.25 × 10^7^ /mL in fresh RPMI 1640 GlutaMAX-I 25 mM HEPES (Gibco) containing 0.4 mM d-luciferin without addition of an oxidative stressor (control) or supplemented with 20 mM hydrogen peroxide (H_2_O_2_; Sigma) or 8 mM tert-butylhydroperoxide (tBOOH; Sigma). The yeasts were incubated at 37°C and 5% CO_2_. The relative light units per second (RLU/s) were measured after 8 h using a TECAN Spark plate reader set at 37°C and 5% CO_2_ in the air space. The assays were performed at least two times in triplicates. The mean of luminescence and standard deviations were plotted. A statistically significant difference between Δ*ku80* and Δku80_Δpks1 was calculated using one-way ANOVA in GraphPad Prism version 9 for Windows.

### Isolation of BMDMs

All animal experiments were approved by the UK Home Office (PPL P6A6F95B5) and University of Exeter Animal Welfare and Ethical Review Body. For isolation of murine bone-marrow from femurs and tibias, we used four 6- to 8-week-old male and female C57BL/6 mice bred in-house at the University of Exeter and housed in stock cages under pathogen-free conditions.

Bone-marrow-derived macrophages were obtained as described previously (66). In brief, bone marrow was extracted from femurs and tibias of C57BL/6 mice, cultured in RPMI 1640 GlutaMAX-I 25 mM HEPES, 100 µg/mL penicillin/streptomycin (abbreviated as RPMI hereafter), and supplemented with 10% (vol/vol) Fetal Bovine Serum (FBS) and 20% (vol/vol) L929-conditioned supernatant on day 1. Medium was replaced on day 4, and cells were used for subsequent experiments on day 7.

### BMDM stimulation and quantification of cytokine production

BMDMs were brought into suspension with PBS containing 8 mg/mL lidocaine and 5 mM EDTA and counted and seeded at 2 × 10^5^ cells per well in 24-well plates in RPMI medium supplemented with 10% FBS. After overnight incubation at 37°C and 5% CO_2_, the medium was replaced with RPMI or RPMI containing 50 ng/mL IFN-γ (Abcam). After 1 h at 37°C and 5% CO_2_, the supernatant was discarded and replaced with fresh RPMI without FBS. Yeasts cultivated in RPMI 2% glucose were centrifuged at 3,220 × *g* for 5 min, washed three times with PBS (Gibco), and counted in a hemocytometer chamber. 2 × 10^7^ yeasts were opsonized in 1 mL RPMI 10% (vol/vol) mouse serum (Sigma) at 37°C for 1 h, centrifuged, washed once in PBS, and resuspended in serum-free RPMI. For cytokine assays, the BMDMs with and without pre-stimulation with IFN-γ were co-incubated with opsonized yeasts at the indicated MOI or with LPS (10 ng/mL) for 3 or 6 h. Supernatant was collected and centrifuged at 4,000 × *g* for 5 min to remove remaining yeast cells, and yeast-free supernatants were kept frozen until cytokine ELISA was performed. The concentration of TNF-α was determined using the DuoSet ELISA kit (R&D systems) according to the manufacturer’s protocol. The statistical analyses were performed using two-way ANOVA and GraphPad Prism version 9 for Windows.

## SUPPLEMENTAL MATERIAL

Supplemental figures and tables spectrum.05078-22-s0001.pdf Figures, table and MM.

ASM does not own the copyrights to Supplemental Material that may be linked to, or accessed through, an article. The authors have granted ASM a non-exclusive, world-wide license to publish the Supplemental Material files. Please contact the corresponding author directly for reuse.
