# Supplementary material for: CRISPR-based tools for targeted genetic manipulation in pathogenic Sporothrix species
Source: Microbiol Spectr. 2023 Sep 14;11(5):e05078-22. doi: 10.1128/spectrum.05078-22 (PMC10581184; doi:10.1128/spectrum.05078-22)
Supplement: Supplemental figures and tables — Figures, table and MM. [file spectrum.05078-22-s0001.pdf]

Supplementary Material

Supplementary Table 1: Oligonucleotides

Number	Sequence	Target	Protocol
1	CTAGAGTCGACCTGCAGCTCGTGCATGTGAAGTTTTCCG	*P. brasiliensis*	Cloning of EF1 promoter, Luc Pb and tENO1 in URA Blaster pUC19
2	TCTTGGCATCCTCCATGTTGAAGAACACAGAACGAATAG	*P. brasiliensis*	Cloning of EF1 promoter, Luc Pb and tENO1 in URA Blaster pUC19
3	ATGGAGGATGCCAAGAACATCAAG	*PbLuc*	Cloning of Pb promoter, Luc Pb and tENO1 in URA Blaster pUC19
4	TCCACGCCCCTATACGGCGATCTTGCCGC	*PbLuc*	Cloning of Pb promoter, Luc Pb and tENO1 in URA Blaster pUC19
5	GCCGTATAGGGGCGTGGAGATGAGATGAG	*P. brasiliensis*	Cloning of Pb promoter, Luc Pb and tENO1 in URA Blaster pUC19
6	GCTTGCATGCCTGCAGCCACTGATGTTGGAGGTGACT	*P. brasiliensis*	Cloning of Pb promoter, Luc Pb and tENO1 in URA Blaster pUC19
7	TTCTAATACGACTCACTATAGCGAGGGACTGGTCTCCAAAGGTTTTAGAGCTAGA	*Sporothrix*	gRNA synthesis for *pks1* deletion
8	TTCTAATACGACTCACTATAGCTACGTCGAGATGCACGGCAGTTTTAGAGCTAGA	*Sporothrix*	gRNA synthesis for *pks1* deletion
9	TGAATTCGAGCTCGGTACCCGGGATGTGTGATGTCTGTCTG	*S. brasiliensis*	Deletion construct *pks1* and probe for Southern blot analysis
10	GCGGCCGCTTATTCTACTTAGCCAGCCTGTCAGCCATG	*S. brasiliensis*	Deletion construct *pks1* and probe for Southern blot analysis
11	TAAGTAGAATAAGCGGCCGCGACGGTACCGAGATGCTGTC	*S. brasiliensis*	Deletion construc*t pks1*
12	GTCGACTCTAGAGGATCCCCGGGCCATTTCGAAGCACGC	*S. brasiliensis*	Deletion construct *pks1*
13	CGTCTTCACTCCAGTCTGTC	*S. brasiliensis*	Control for deletion construct *pks1*
14	GCCTGTCTCCATTGCTTCG	*Sporothrix* sp.	Deletion control of *pks1* (upstream)
15	GAT GCC GTT GGC ACT GGA TG	*Sporothrix* sp.	Deletion control of *pks1*-coding region (upstream)
16	GCG CCA AGA CAG AGC TCA C	*Sporothrix* sp.	Deletion control of *pks1*-coding region (downstream)
17	CCA GCG TCT CAA AGT CGT C	*Sporothrix* sp.	Deletion control of *pks1* (downstream)
18	CGG TAT CGG TAG GCG GTG	*nat1*	Deletion control of *pks1-nat1* construct (upstream)
19	GGGTTTCACCCTCTGTGGTC	*nat1*	Deletion control of *pks1-nat1* construct (downstream)
20	TTCTAATACGACTCACTATAGGCGTCGCATTGCGGGCAAGAGTTTTAGAGCTAGA	*Sporothrix* sp.	gRNA synthesis for *ku80* deletion
21	TTCTAATACGACTCACTATAGTCTCGAGCTGCGTAATGTTGGTTTTAGAGCTAGA	*Sporothrix* sp.	gRNA synthesis for *ku80* deletion
22	TTCTAATACGACTCACTATAGACAGATGGGCCAGATTGTGGGTTTTAGAGCTAGA	*Sporothrix* sp.	gRNA synthesis for *ku80* deletion
23	TTCTAATACGACTCACTATAGTCGGCGCCTAGCACCAAAGAGTTTTAGAGCTAGA	*Sporothrix* sp.	gRNA synthesis for *ku80* deletion
24	GAATTCGAGCTCGGTACCCGGGTTCATTGCTCAGTATGGTACAG	*S. schenckii*	Deletion construct of *ku80* and probe for Southern blot analysis
25	TTCACATGCACGAGAGGCCTCGTCCGTCACAAGCACAATC	*S. schenckii*	Deletion construct of ku80 and probe for Southern blot analysis
26	GCTTGTGACGGACGAGGCCTCTCGTGCATGTGAAGTTTTCCG	*P. brasiliensis*	Deletion construct of *ku80* (luciferase construct)
27	CGGGTCGCGGCCGCAGGCCTCCACTGATGTTGGAGGTGACT	*P. brasiliensis*	Deletion construct of *ku80* (luciferase construct)
28	CAGTGGAGGCCTGCGGCCGCGACCCGAGTCAGACATGCG	*S. schenckii*	Deletion construct of *ku80*
29	GTCGACTCTAGAGGATCCCCGGGCCATGAGCAACCCG	*S. schenckii*	Deletion construct of *ku80*
30	GATGTCCAATTGCTCACTCTAC	*Sporothrix* sp.	Deletion control of *ku80* (upstream)
31	CAT CGA AGT CGA CAC CGC TG	*Sporothrix* sp.	Deletion control of *ku80* -coding region (upstream)
32	CGGTCCTACCATCAAGAAGG	*Sporothrix* sp.	Deletion control of *ku80*-coding region (downstream)
33	CAG ATA ACA ACT TGC TGT CCA G	*S. brasiliensis*	Deletion control of *ku80* (downstream)
34	GCT AGC AGA TAT TGA CTT GTT GTC	*S. schenckii*	Deletion control of *ku80* (downstream)
35	GGAGCTCGACAACAAGGGTC	*P. brasiliensis*	Deletion control of *ku80*-luciferase construct

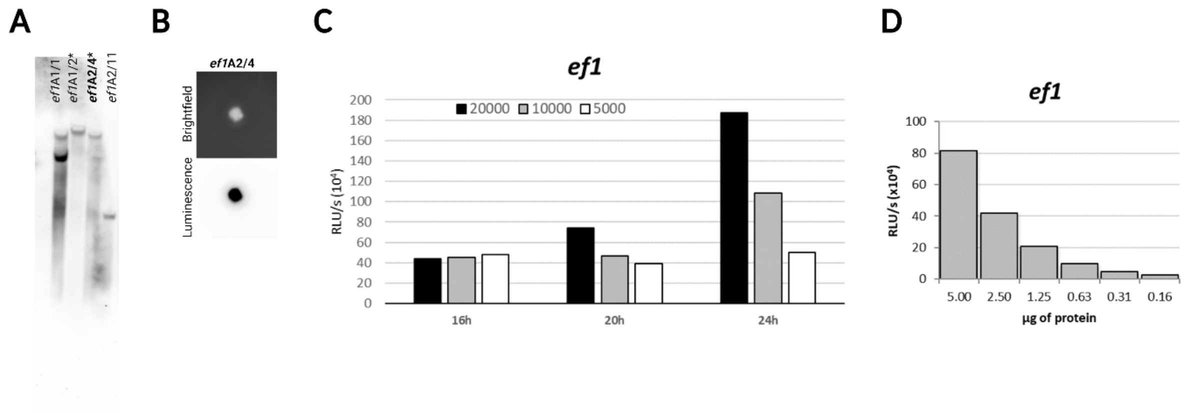

Supplementary Figure 1: Luciferase construct for expression in *Sporothrix* species. The efficiency of the plasmid harbouring *PbLuc* under the control of *elongation factor 1-gamma* (*ef1*) promoter from *Paracoccidioides* was tested in *A. niger* transformation. **(A)** *A. niger* transformant containing single integration of the plasmid was selected by Sothern blot analysis. **(B)** 500 conidia from *A. niger PbLuc* transformant containing single integration of the plasmid were spotted in GG10 containing D-luciferin (0.2 mM). Brightfield (upper panel) and luminescence (bottom panel) were recorded after 2 days of incubation at 28 ºC. **(C)** *In vivo* and **(D)** *in vitro A. niger* assay for *PbLuc* expression under the control of *ef1* promoter.

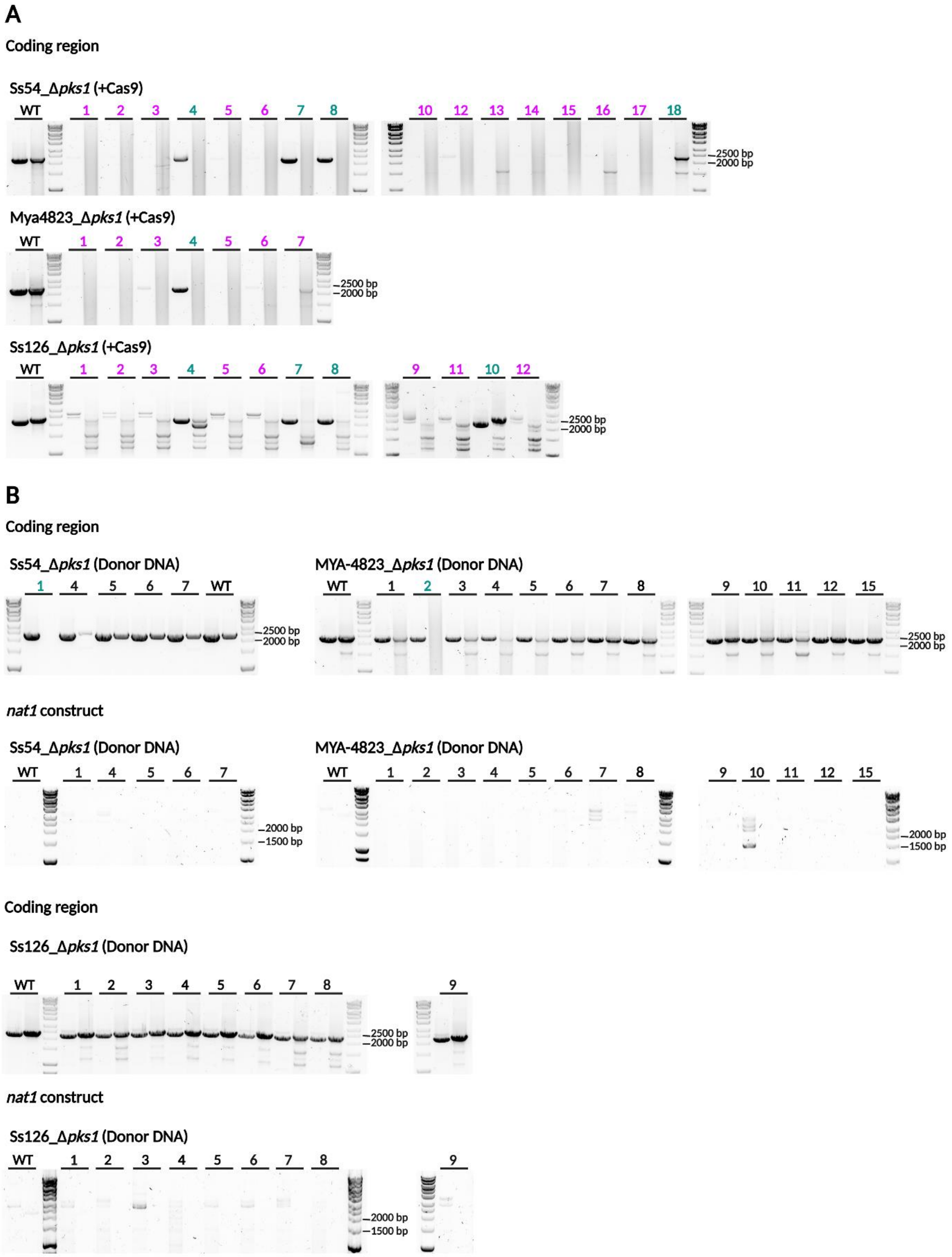

Supplementary Figure 2: Validation of *pks1* disruption. **(A)** PCR analysis for identifying the disruption of *pks1* coding region using the gDNA from 16 Ss54 colonies, 7 MYA-4823 colonies and 12 Ss126 colonies from transformations performed in the presence of Cas9. **(B)** PCR analysis for identifying the disruption of *pks1* coding region and *nat1* cassette insertion using the gDNA from 5 Ss54 colonies, 13 MYA-4823 colonies and 9 Ss126 colonies generated in the absence of Cas9. For each transformant the left-hand lane represents PCR diagnosis of the *pks1* deletion or *nat1* insertion at the upstream gRNA site, and the right-hand lane the diagnosis of the *pks1* deletion or nat1 insertion at the downstream gRNA site. Strains with the WT coding region (black) yield PCR products at 2257 bp and 2337 bp. For transformations with Cas9, strains containing one PCR product (green) or double negative (purple) for the presence of *pks1* coding region were further analysed for the integration of deletion cassette in the Figure 2C.

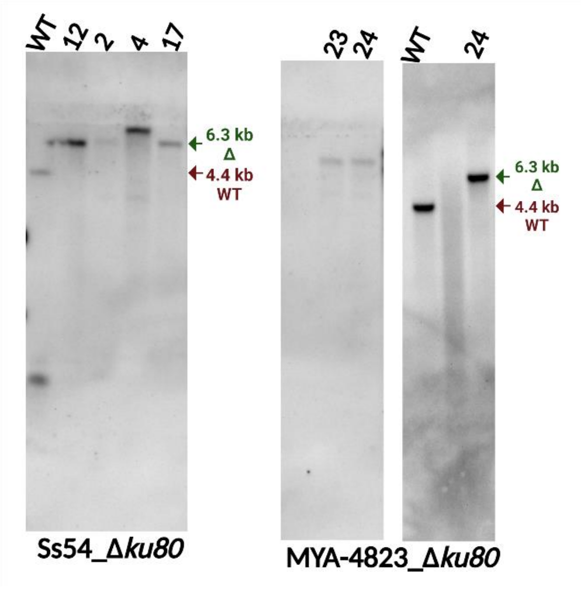

Supplementary Figure 3: Southern blot analysis of *ku80* deletion strains. Southern blotting showing 4 and 2 transformants from Ss54 and MYA-4823 containing in locus integration. Diagnostic bands of 4.4 kb for wild type (WT) and 6.3 kb for a deletion mutant (Δ) are highlighted.

Supplementary Methods: *Aspergillus* transformation and luciferase assay. *Aspergillus niger* A1144 Δ*pyrG* (1) was cultivated at 28 °C in modified *Aspergillus* Minimal Medium-GG10 (2) supplemented with 10 mM uridine and 2% (w/v) agar when required. For DNA extraction and luciferase activity assay, *A. niger* was cultured overnight in YPD medium. A1144 Δ*pyrG* conidia were harvested from slants in Phosphate-Buffered Saline (PBS) with 0.1% (v/v) Tween 20, washed twice with PBS and the conidial concentration determined by counting in a haemocytometer chamber. The protoplast-mediated transformation was performed as previously described, using p*ef1*:*Pbluc*$_{OPT\_red}$:t*eno1*_URABlaster_pUC19 (1-3). To identify transformants containing single integration of the plasmid, we performed Southern blot analyses of the *Eco*RI digested gDNA for the detection of *PbLuc* as previously described (3). 500 conidia of the transformant *ef1*A2/4 from *Aspergillus niger* was spotted in GG10 agar supplemented with 0.4 mM of D-luciferin and incubated at 28 °C for 2 days. The bioluminescence was recorded using a ChemiDoc XRS+ system (Bio-Rad). The *PbLuc* activity was measured in crude cell-free extracts and *in vivo* as described by (3).

References

1. Geib E, Baldeweg F, Doerfer M, Nett M, Brock M. 2019. Cross-Chemistry Leads to Product Diversity from Atromentin Synthetases in Aspergilli from Section Nigri. Cell Chem Biol 26:223-234 e6.
2. Geib E, Gressler M, Viediernikova I, Hillmann F, Jacobsen ID, Nietzsche S, Hertweck C, Brock M. 2016. A Non-canonical Melanin Biosynthesis Pathway Protects Aspergillus terreus Conidia from Environmental Stress. Cell Chem Biol 23:587-597.
3. Milhomem Cruz-Leite VR, Tomazett MV, Santana de Curcio J, Sbaraini N, Bailao AM, Goncales RA, Moraes D, Pereira M, Vainstein MH, Schrank A, Peres da Silva R, Brock M, Maria de Almeida Soares C. 2022. Bioluminescence imaging in Paracoccidioides spp.: a tool to monitor the infectious processes. Microbes Infect 24:104975.
